# Spontaneous Resolution of Early-Onset Pediatric Trigger Thumb: A Case Study

**DOI:** 10.7759/cureus.80966

**Published:** 2025-03-21

**Authors:** Simonne M Jones, Brett F Shannon, Chabelly Gomez, Katie Mayer, Naomi G Jury

**Affiliations:** 1 Surgery, St. George's University School of Medicine, St. George's, GRD; 2 Orthopedic Surgery, Nemours Children’s Clinic, Orlando, USA; 3 Family Medicine, Keralty Hospital, Miami, USA; 4 Psychiatry and Behavioral Sciences, St. George's University School of Medicine, St. George's, GRD; 5 Plastic Surgery, Miami Dade College, Miami, USA

**Keywords:** congenital hand surgery, pediatric hand surgery, pediatric trigger thumb, trigger thumb management, trigger thumb surgery

## Abstract

Pediatric trigger thumb (PTT) is a deformity in the flexion of the thumb due to stenosing tenosynovitis. Surgery is the standard treatment protocol when the thumb is held in fixed flexion after 24 months of age. However, emerging research provides evidence that cases with a fixed deformity can be resolved without surgical management beyond 24 months. The patient, in this case, was a four-year-old female diagnosed with congenital pediatric trigger thumb with early onset in infancy starting at four months. She displayed complete resolution and release of the flexion deformity without surgical treatment at 40 months. It is possible that non-invasive treatment on fixed flexion, such as splinting and observation, extending beyond 24 months before proceeding with surgical intervention should be considered. While the mechanism is only hypothesized, resolution may occur through a combination of tissue remodeling, tendon adaptation, and gradual reduction of the entrapment. Waiting for the potential release of the A1 pulley to occur spontaneously, even in patients with a fixed deformity, may be a successful treatment modality.

## Introduction

Pediatric trigger thumb (PTT) is considered a deformity that typically presents in the first two years of life. It presents as a flexion contracture of the interphalangeal (IP) joint where stenosing tenosynovitis constricts and thickens the A1 pulley, ultimately locking the thumb in a fixed flexed position. The standard treatment for PTT has been identified as surgery when the joint can no longer be extended and is often performed before age three in cases of stage III and IV PTT. It has been reported that spontaneous resolution can occur without surgery in 43.5% of patients with this condition, and non-surgical interventions may better equip parents and surgeons with informed treatment options. This occurs through a combination of tissue remodeling, tendon adaptation, and reduction of the entrapment [1].

There is conflicting data when it comes to research that has reported the likelihood of spontaneous release of PTT. Baek and Lee reported that 75.9% of 87 thumbs in 67 patients with an average age of two years displayed spontaneous resolution of the flexion deformity, including fixed and non-fixed deformity [2]. However, a recent study by Chew et al. reported that in the same age group, 37% of 62 patients presenting with 79 thumbs locked in flexion had spontaneous resolution [3]. The inconsistencies among earlier studies reveal that the patient population is challenging to investigate, perhaps due to variable cohorts or parents continuing observation and non-invasive management without immediate signs of improvement.

Research published among European Pediatric Orthopedic Society members characterizes cases of stage IV pediatric trigger thumb with fixed flexion deformity as having a low probability of resolving without surgery [4]. When considering the outcomes long term, only 33% of patients showed resolution of the A1 pulley without surgical intervention, which justifies the recommendation by physicians to initiate surgical management with the release of the pulley rather than watchful waiting for extended periods of time [5]. Researchers observed that for every increase in the degree of the angle of initial IP joint flexion, there was a 3% decrease in the probability of spontaneous resolution. 

Identifying the presence of a nodule called “Notta’s nodule” on physical examination combined with an assessment of the severity of the deformity of the joint has been used as a prognostic factor when considering treatment options for trigger thumb, as the presence of a Notta’s nodule can indicate a longer period of tendon entrapment. A study by Rodgers and Waters reports that the severity of the deformity can help inform clinical decision-making when there is uncertainty on whether to treat PTT conservatively or with surgery. This is especially prevalent in cases where the patient is older than the period in which most are known to resolve spontaneously [6]. The time frame in which physicians recommend surgery to patients may warrant more investigation. Due to emerging evidence and studies of cases that contradict surgical intervention, the necessity of surgery over an observation period beyond 24 months in pediatric trigger thumbs with a stage IV fixed flexion deformity may need reconsideration. Less invasive or conservative treatments such as observation and splinting may still be offered as they reduce risk and may still produce favorable results [7].

## Case presentation

**Figure 1:**
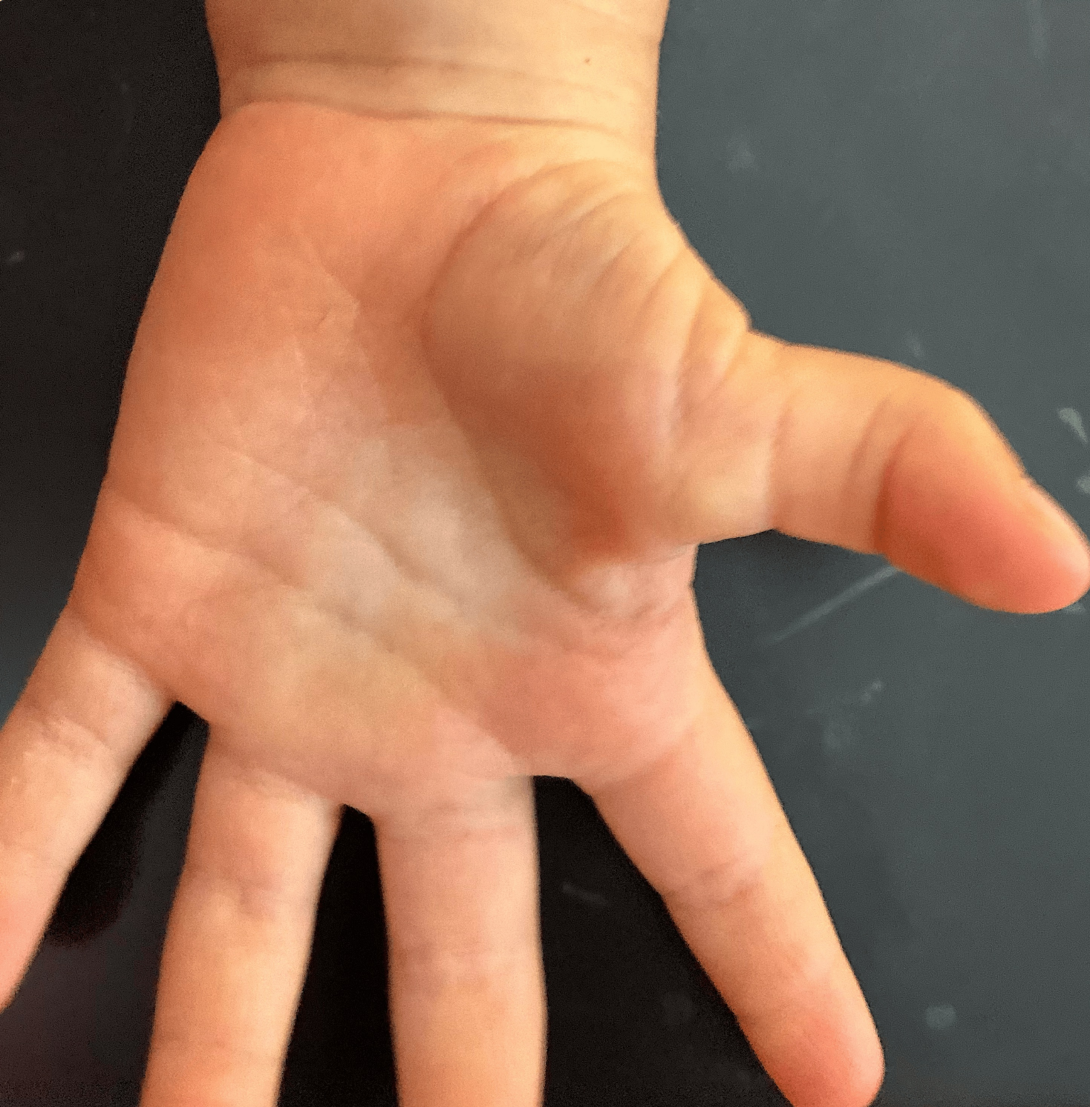
Preoperative clinical photo of a 22-month-old patient with a left thumb interphalangeal joint flexion deformity due to a chronic pediatric trigger finger. The image demonstrates loss of full extension secondary to stenosing tenosynovitis.

The patient was a four-year-old female with a history of PTT who first presented with a stage 1 deformity at four months of age. The patient later presented to the pediatric office with her parents due to a persistent flexion or a stage IV deformity of the left thumb IP joint for the past 39 months. The patient’s parents cited an inability to extend the thumb IP joint passively or voluntarily. For the first nine months of life, the thumb IP joint would fully extend intermittently but then would return to the fixed position. By nine months of age, the joint became permanently locked in flexion, resulting in a complete loss of extension that persisted thereafter (Figure 1).

The patient’s parents reported no history of trauma, infections, or major childhood illness. On examination over several appointments, a nodule was palpated at the A1 pulley and was clinically determined to be fibrotic tissue or Notta’s nodule with a classic flexion contracture. The thumb could not be actively or passively extended. After a clinical diagnosis of a stage IV deformity was made, surgical treatment was recommended. Due to the severity of the PTT, no imaging modality was necessary for diagnosis.

**Figure 2:**
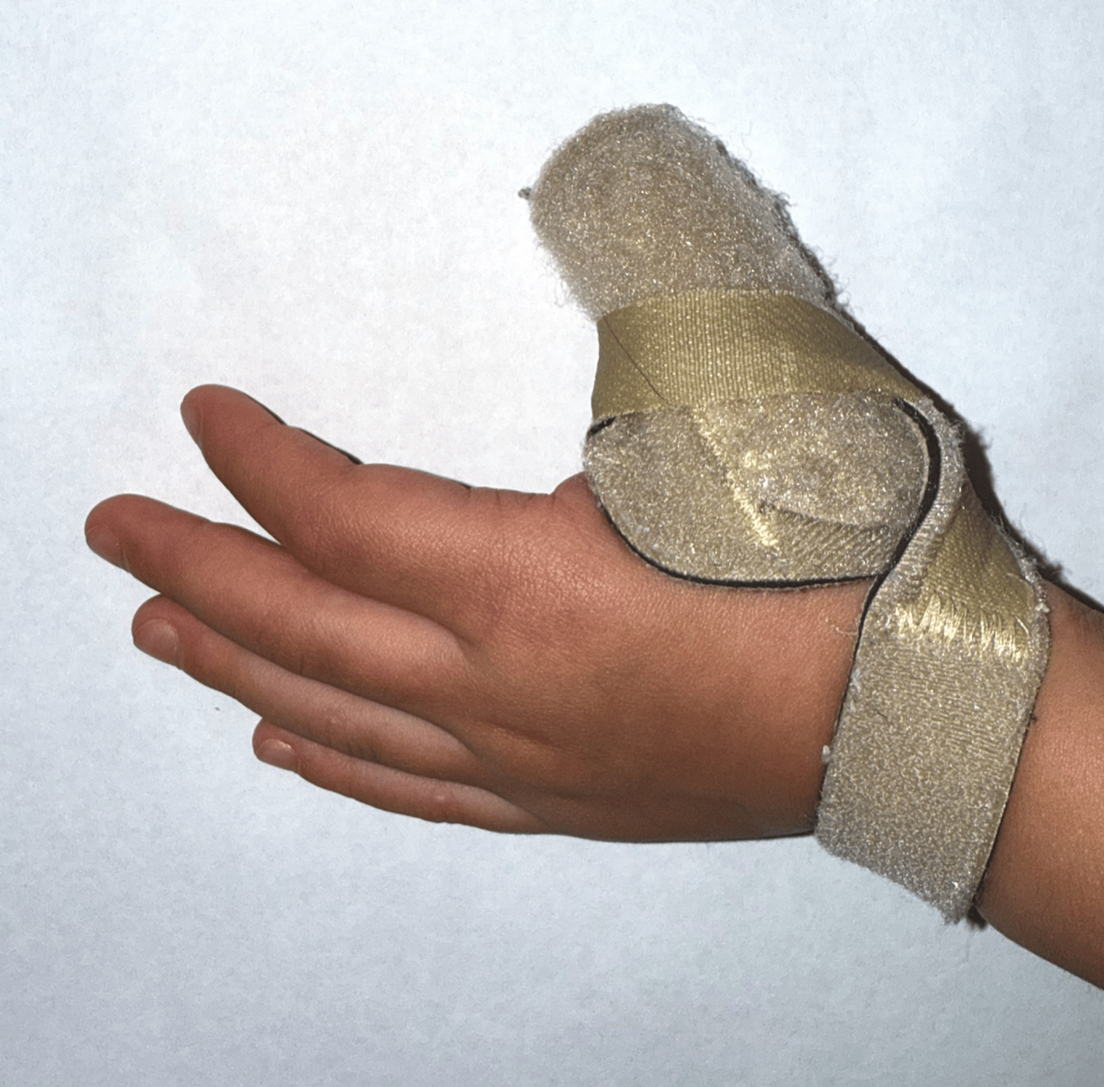
The patient is wearing a splint designed to maintain the thumb in extension. Splinting was initiated to improve joint mobility and prevent locking of the flexion deformity.

The patient was scheduled for outpatient trigger finger release surgery. During the waiting period, at 40 months, the child experienced a single episode of full active extension for two hours before locking back into flexion. This was the first episode of this nature since nine months of age. The parents noted a second episode two weeks later, and immediately following this, a clinician advised a splint. The splint was used by the patient for two weeks consistently, then nightly for approximately one month (Figure 2). No other non-invasive treatments, such as stretching or injections, were used.

**Figure 3:**
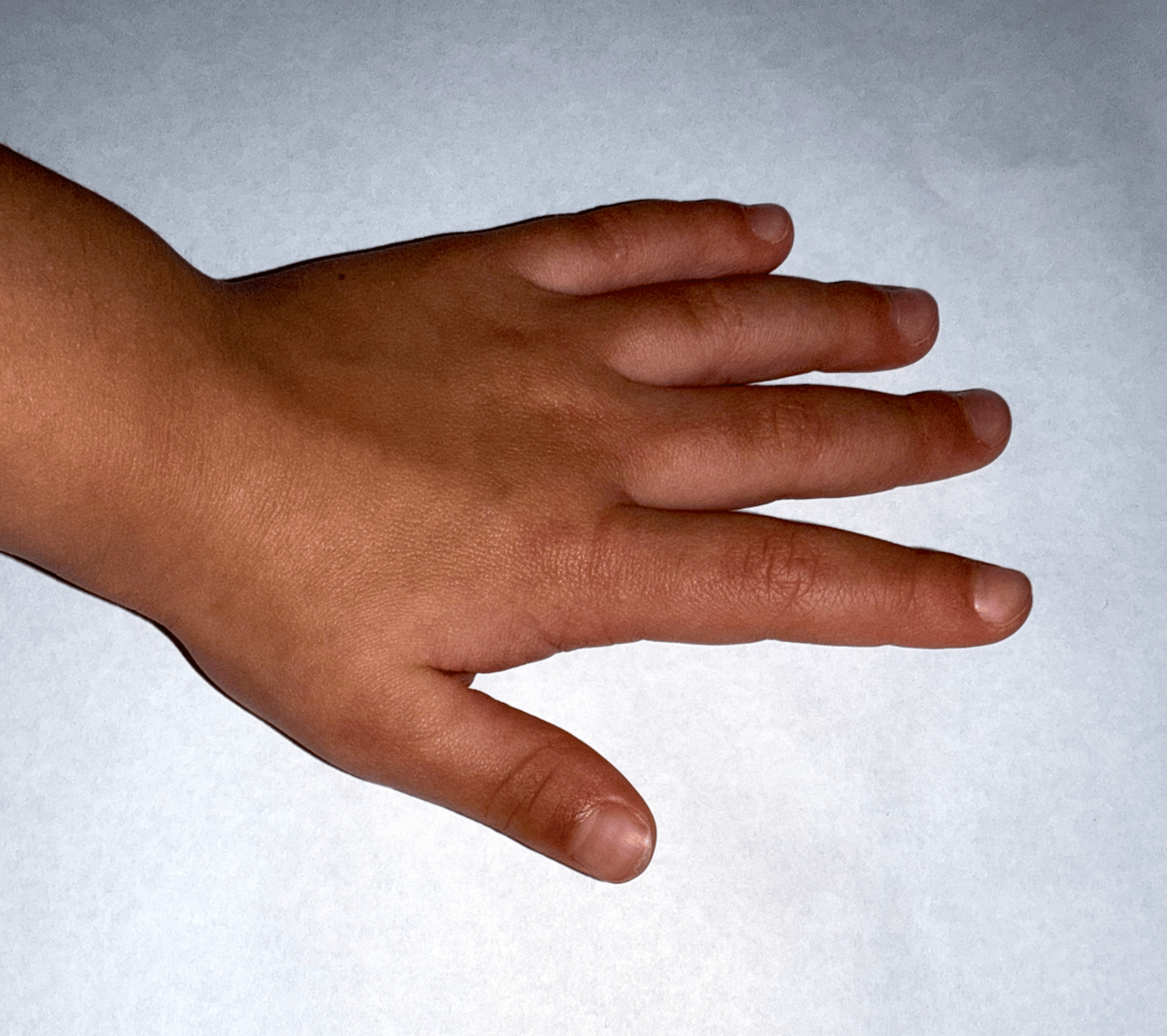
Clinical photo of the patient at four years of age with complete resolution of the fixed trigger thumb, demonstrating full active extension of the interphalangeal joint without residual deformity. No surgical intervention was performed, highlighting the potential for spontaneous resolution in early-onset pediatric trigger thumb.

At the six-month follow-up, parents reported complete improvement in thumb mobility. By the 12-month mark, complete resolution of the flexion contracture was observed, with full active extension and no palpable nodule. The child regained normal thumb function without requiring surgical intervention (Figure 3). 

## Discussion

This study demonstrates a case of PTT first documented at four months of age and locked in stage IV fixed flexion for three years before spontaneous resolution. While this resolution could have been an outlier, splinting may have played a role in maintaining joint stability. A flexion deformity released without medical intervention beyond the standard age documented by research may help in informing surgical care. Based on prior research, the patient would typically be considered a candidate for surgical intervention. Studies that have guided treatment protocols, such as the study by Baek and Lee, showed that patients with milder cases have higher chances of PTT resolving naturally without surgery [2]. Since the likelihood of PTT resolving spontaneously decreases with age, especially in children older than 24 months, surgery is highly recommended by surgeons [3]. The recommended treatment for PTT when non-invasive treatment has failed is open surgery for children, and newer approaches involve percutaneous release with a 90% success rate [8]. However, emerging research has also shown that children over the age of 24 months can still have spontaneous resolution if given adequate time for observation. A meta-analysis found that 58.9% of pediatric trigger thumb cases resolved spontaneously after 24 months of follow-up, which further highlights the potential benefits of extended observation before considering surgical intervention.

**Figure 4:**
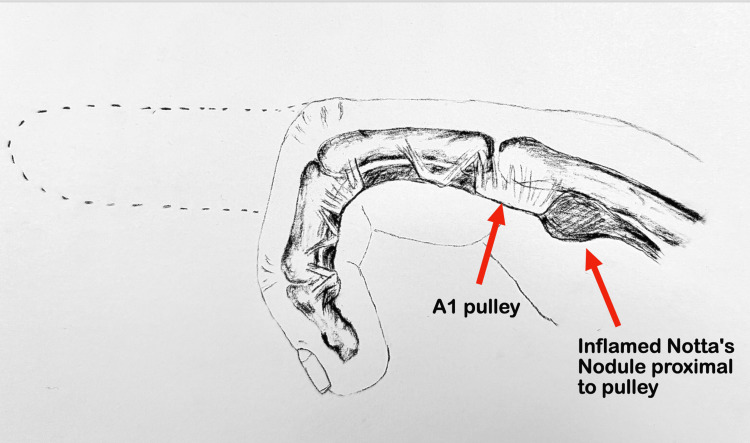
The anatomy of congenital trigger thumb, illustrating an inflamed Notta’s nodule trapped proximal to the A1 pulley. The thickened flexor pollicis longus tendon locks the digit in flexion. Image credits: Simonne Jones

In this case, the patient displayed the most severe presentation possible: a stage IV deformity, defined by the flexion deformity being permanently locked where the IP joint is unable to be passively extended. In this case, it was locked proximal to the A1 pulley, resulting in fixed flexion. Since the patient was already presenting as stage IV at nine months of age without improvement, they were a good candidate for surgical release (Figure 4). The parents were told there was no other treatment option in order to avoid permanent deformity and functional impairment. A study by Hutchinson et al. recommends delaying surgery beyond the established three years of age along with a year of observation, even in the case of severe fixed flexion contracture [5]. In recent studies, delaying surgery to observe the patient for longer periods of time is usually reserved for patients who can passively extend the joint [1, 3]. However, in this case of locked flexion, the patient experienced full resolution of the trigger thumb without any invasive treatment or surgery. Tang et al. note that a large portion of PTT cases can resolve in patients older than the age of 24 months [1]. Perhaps cases with more severe deformities, such as this patient, can also resolve without surgery if offered a longer waiting period and alternatives to surgery. 

This case challenges the established treatment approach and suggests that longer observation time is a viable consideration for treatment before committing to surgical intervention. Studies like the one by Kikuchi and Ogino and Slakey and Hennrikus support the use of less invasive treatment modalities such as splinting [9, 10]. Yano et al. conducted research that reported no difference between outcomes in patients who received surgery vs. splinting for PTT [11]. In this case, a splint could have played a key role in the outcome, which supports conservative management.

To fully understand the factors contributing to the spontaneous resolution of persistent flexion contracture, more information and research are needed. Conservative treatments like splinting warrant further investigation. Delaying surgery while continuing observation longer than 24 months is supported, considering that delayed surgical treatment does not cause permanent disability or deformity. A study on trigger thumb in children who had surgical treatment above five years of age showed no postoperative deformities or complications [12].

Treatment releasing the A1 pulley surgically if the patient has remained in a stage IV contracture for several years without being able to passively extend the joint is still the gold standard for severe cases. Our case study aligns with research supporting conservative treatment options and a longer period of continued observation in patients with stage IV deformity.

## Conclusions

In summary, pediatric trigger thumb resulting in persistent flexion at the IP joint is commonly treated surgically before age three. For patients with a stage IV deformity where the IP joint is locked in contraction and cannot be passively extended, there is still potential for spontaneous resolution naturally. Our case study supports extending the age criteria for observation beyond 24 months in fixed flexion trigger thumbs as first-line treatment. Given the opportunity for spontaneous resolution with extended observation and the lack of permanent deformity in patients who delay invasive intervention, surgical management may be avoided even in cases of stage IV deformity. Further investigation is needed to better identify the causes of spontaneous resolution and examine the role of splinting in conservative management.
